# Chimpanzees Help Each Other upon Request

**DOI:** 10.1371/journal.pone.0007416

**Published:** 2009-10-14

**Authors:** Shinya Yamamoto, Tatyana Humle, Masayuki Tanaka

**Affiliations:** 1 Primate Research Institute, Kyoto University, Inuyama, Aichi, Japan; 2 Wildlife Research Center, Kyoto University, Sakyo-ku, Kyoto, Japan; University of Liverpool, United Kingdom

## Abstract

**Background:**

The evolution of altruism has been explained mainly from ultimate perspectives. However, it remains to be investigated from a proximate point of view how and in which situations such social propensity is achieved. We investigated chimpanzees' targeted helping in a tool transfer paradigm, and discuss the similarities and differences in altruism between humans and chimpanzees. Previously it has been suggested that chimpanzees help human experimenters by retrieving an object which the experimenter is trying to reach. In the present study, we investigated the importance of communicative interactions between chimpanzees themselves and the influence of conspecific partner's request on chimpanzees' targeted helping.

**Methodology/Principal Findings:**

We presented two tool-use situations (a stick-use situation and a straw-use situation) in two adjacent booths, and supplied non-corresponding tools to paired chimpanzees in the two booths. For example, a chimpanzee in the stick-use situation was supplied with a straw, and the partner in the straw-use situation possessed a stick. Spontaneous tool transfer was observed between paired chimpanzees. The tool transfer events occurred predominantly following recipients' request. Even without any hope of reciprocation from the partner, the chimpanzees continued to help the partner as long as the partner required help.

**Conclusions/Significance:**

These results provide further evidence for altruistic helping in chimpanzees in the absence of direct personal gain or even immediate reciprocation. Our findings additionally highlight the importance of request as a proximate mechanism motivating prosocial behavior in chimpanzees whether between kin or non-kin individuals and the possible confounding effect of dominance on the symmetry of such interactions. Finally, in contrast to humans, our study suggests that chimpanzees rarely perform acts of voluntary altruism. Voluntary altruism in chimpanzees is not necessarily prompted by simple observation of another's struggle to attain a goal and therefore an accurate understanding of others' desires in the absence of communicative signals.

## Introduction

What is similar and what is different in altruism between humans and non-human animals? Previous studies have provided theoretical explanations for the evolution of altruism [1], [2], and have found evidence for altruistic behavior in a range of animal species [3], [4]. There is little doubt that humans share something about altruism with non-human animals. However, we still know little about how such social propensity is proximally elicited. While some suggest that empathy is one of the underlying mechanisms for altruism [5], others have argued that selfish motivations, e.g. harassment avoidance, best explain apparently altruistic behavior in non-human animals [6]. By investigating what kind of situations and what factors may favor altruistic behavior, we may be able to shed further light on the proximate mechanisms underlying altruistic behavior across taxa. In order to investigate the similarities and differences between humans and non-human animals, experimental studies under controlled conditions provide a valuable tool to investigate proximate mechanisms.

Recent studies have revealed that common marmosets [7] and capuchin monkeys [8]–[9] show prosociality, i.e., spontaneously sharing food with non-reciprocating and genetically unrelated individuals. However, controversy still abounds as to whether or not chimpanzees, one of our evolutionary closest living relatives, also show such other-regarding preferences. Observations in the wild and captivity have reported evidence of altruism and cooperation in chimpanzees, such as food sharing, grooming, coalition formation, consolation and cooperative hunting [10]–[13]. Warneken and his colleagues have also experimentally demonstrated that chimpanzees can help a human and conspecific partner without any benefit to themselves [14]–[15]. Meanwhile however, other experimental studies concerning prosociality in chimpanzees have mostly produced negative results. When chimpanzees were offered a choice between two options, a mutually beneficial option or a selfishly beneficial option, they did not change their choice whether a conspecific partner was present or absent [16]–[18]. From these results, it has been suggested that chimpanzees are not other-regarding.

Two possible proximate factors influencing altruism in chimpanzees have been highlighted: recipient's request and the presence of food [14]–[15; for review see 19]. Negative results were produced from experimental setups employing food rewards in a choice paradigm [16]–[18], while positive results emanated from experiments in which food was not presented as a direct reward to the participating chimpanzees [14]–[15]. Warneken et al. [14] also revealed that chimpanzees handed an object to a human experimenter more frequently when the experimenter exhibited request than when the experimenter did not. These studies have highlighted the possible importance of recipient's request upon altruistic behavior in chimpanzees; however, systematic examination and analysis of the role of communicative interactions between chimpanzees themselves in other regarding, possibly altruistic contexts is still lacking.

In the present study, we examined the influence of a conspecific recipient's request on helping behavior in chimpanzees, using an experimental paradigm involving tool transfer rather than food transfer. This experimental paradigm was similar to that developed by Savage-Rumbaugh, Rumbaugh, and Boysen [20], which investigated tool transfer mediated by symbolic communication between chimpanzees. In their experiment, however, the symbolic communication, the tool transfer and the following food sharing were all artificially trained by human experimenters. We decided to replicate this kind of test paradigm without training in order to examine whether or not tool transfer spontaneously occurs. Assuming that the helper incurs a minimal energetic cost in transferring the tool to his or her conspecific partner and considering that the helper gains no direct immediate personal benefit in performing such kind of targeted helping, we consider tool transfer in this context to be altruistic.

One of the important points of the present study is that we analyzed interactions between conspecific partners, and not between a chimpanzee and a human partner. In a cooperation task in which simultaneous rope-pulling by two individuals produced rewards to both participating subjects, a chimpanzee solicited a familiar human partner but not a conspecific partner [21]. These results have suggested that such chimpanzees' communicative interactions are specific to contexts involving human partners but not conspecific partners. In the context of this paradigm, this may be explained by differences in the social relationship between conspecific and non-conspecific dyads. In contrast to that characterizing chimpanzees, the relationship between a human experimenter and a chimpanzee is generally characterized by absence of competition, as well as reinforcement of compliant behavior, two factors potentially favoring chimpanzees' motivation to help even if unrewarded or solicit help when required [14]. Meanwhile, communicative interactions involving request between chimpanzees has also been reported in an experiment requiring chimpanzees to insert tokens in turn providing rewards to their partner [22]. In this experiment, chimpanzees on occasion exhibited request toward their conspecific partner, and the partner responded to the request by inserting a token, although these interactions failed to result in continuous turn taking behavior between the two partners.

We investigated the importance of request in chimpanzees' helping behavior involving the transfer of a tool from both the point of view of the donor and the recipient. In experiment 1, we examined whether or not chimpanzees transfer a tool required by their partner to obtain a juice reward, and how influential a recipient's request is upon the donor's helping behavior. In experiment 2, another possible influential factor, reciprocity, was examined; that is, we evaluated which, short-term reciprocity or response to request, is more salient as a proximate mechanism for altruism in chimpanzees.

## Methods

### Participants

Participants were socially housed chimpanzees at the Primate Research Institute, Kyoto University (KUPRI). All participants had previously taken part in a variety of perceptual and cognitive studies [23]–[24], including social tasks involving contexts involving food sharing [25], token sharing [26], and reciprocity [22], [27]–[28]. The test paradigm for this study was, however, novel to the participants. The participants spend their daily life with other group members in enriched facilities [29], and had ad libitum access to water and were not food deprived. The present study was approved by the Animal Care Committee of the Primate Research Institute of Kyoto University, and the chimpanzees were tested and cared for according to “the Guide for the Care and Use of Laboratory Primates, 2^nd^ edition” produced by the ethics committee of the Primate Research Institute of Kyoto University (2002).

Six pairs of chimpanzees voluntarily participated in this study; three mother-offspring pairs (Ai-Ayumu, Chloe-Cleo, and Pan-Pal; in the order of mother-offspring) and three non-kin adult female pairs (Pendesa-Puchi, Pendesa-Mari, and Puchi-Mari; in the order of dominant-subordinate). The three offspring were born in 2000 at KUPRI, and were raised by their biological mothers in a community of 14 chimpanzees. They were 7 years old at the start of testing. At that time, all three offspring were more and more independent of their mother; for example, they sometimes participated in experiments, separated from their mother. For this study, however, they always came to the experimental room with their mother, since the experimenter called upon both of them to participate in this study.

The chimpanzees at KUPRI have had some experience with tool-using in their ordinary life as well as in some experiments, including stone-use for cracking nuts [30] and twig-use for dipping honey [31]–[33]. For the present study, two novel tool-use situations, i.e., straw-use for drinking juice and stick-use for reaching a juice container, were developed. All participants to this study became experts at these two novel tasks after some training.

### Apparatus and setup

**Figure 1:**
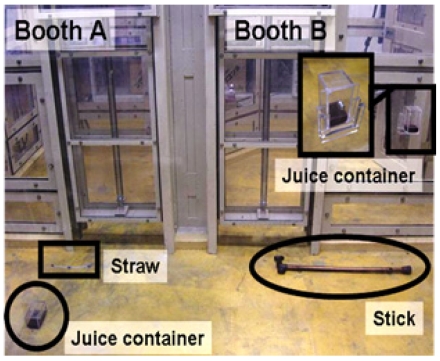
The apparatus and setup for experiment 1: view from outside the booths. In this picture, the stick-use situation was set up in booth A, and the straw-use situation was set up in booth B. A straw was supplied to booth A, but could be effectively used only by the chimpanzee in booth B. A stick was supplied to booth B, but could be effectively used only by the chimpanzee in booth A. In the actual procedure, the tools (a straw and a stick) were supplied in the booths (not presented outside the booths as depicted here), and the partition of booth B was closed (not partially opened as depicted here).

We developed an apparatus and setup for the “tool transfer task” in an experimental room (Figure 1). The testing paradigm required the chimpanzees to obtain a tool which the partner possessed. We expected spontaneous tool transfer between chimpanzees, and analyzed how tools were transferred. The chimpanzee participants were tested in two adjacent booths (136 cm×142 cm and 155 cm×142 cm, 200 cm high). The walls and partitions consisted of transparent polycarbonate panels. We made a hole (12.5 cm×35 cm) in the panel between the two booths. Chimpanzees could thus transfer tools or poke their arm or hand through this hole, but could not reach for a tool on the floor of the adjacent booth, since the hole was placed approximately 1 m above the floor.

We developed two tool-use situations: a stick-use situation in which a stick was necessary for drawing in a juice container placed outside the booth, and a straw-use situation in which a participant needed a straw for drinking juice. In the stick-use situation, a juice container was set on the floor out of the partner chimpanzee's reach. The participant could draw in the juice container with a stick (45 cm long). In the straw-use situation, a juice container harboring a small hole (1 cm in diameter) was fixed to the wall of the booth. A participant could drink the juice through a straw (18 cm long and 8 mm in diameter). Since the walls consisted of transparent panels, a participant could see not only his/her own situation but also the situation of his or her partner in the adjacent booth. We used grape juice (50 ml per individual per trial) for reward.

### Procedure: Experiment 1

We called in a pair of chimpanzees from the outdoor enclosure, and led them one by one into the separate booths. After getting them inside the booths, we set up each tool-use situation for each participant. We developed two conditions: a “matched condition” and a “mismatched condition”. In the matched condition, an appropriate tool was supplied to a matched tool-use situation; that is, a straw was supplied to a participant in the straw-use situation, and a stick was supplied to a participant in the stick-use situation. In contrast, in the mismatched condition, a straw was supplied to a participant in the stick-use situation, and a stick was supplied to a participant in the straw-use situation. In this mismatched condition, both participants had to obtain the tool located in the adjacent booth in order to access the juice reward available to them in their respective booth.

A trial started as soon as we supplied tools to both participants, and lasted for 5 minutes whether or not the participants succeeded in getting the juice reward. We conducted 2 trials (the two tool-use situations for each participant) during a daily session for each pair. The order of the two tool-use situations was counterbalanced across trials. After every three mismatched condition sessions, we conducted a matched condition session. In sum, each pair experienced 24 trials (12 daily sessions) of the mismatched condition (12 trials of each tool-use situation for each participant), and 8 trials (4 daily sessions) of the matched condition (4 trials of each tool-use situation for each participant).

### Procedure: Experiment 2

The aim of experiment 2 was to investigate the mechanism which maintained bilateral mutual helping. Two mechanisms were considered: exchanging reciprocally or just responding to each other's request. In order to examine whether or not bilateral tool transfer was maintained in a short-term reciprocal manner, we tested the mothers and offspring in a situation in which they could not expect any short-term reciprocation from the partner. The non-kin pairs were not tested in experiment 2, since targeted helping in the non-kin pairs as revealed in experiment 1 was already unilateral, which suggested that reciprocity was not the main mechanism for perseverance of tool transfer in the non-kin pairs.

In experiment 2, we assigned each individual (mother or offspring) of a pair as a giver or as a recipient, and fixed the roles consecutively for 24 trials (approximately one week). In the first 24 trials, the mothers were assigned as givers, and the offspring were assigned as recipients. We set up either tool-use situation (the stick-use situation or the straw-use situation) in the offspring's booth, and supplied a corresponding tool in the mother's booth. Thereafter, after an interval of more than one week without tests, the roles were reversed in the second series of 24 trials; the offspring were assigned as givers, and the mothers as recipients. A trial started when we supplied a tool to either participant, and lasted for 5 minutes whether or not tool transfer was observed. We conducted 4 trials (2 trials for each of the two tool-use situations) for each pair in a daily session. The order of the two tool-use situations was counterbalanced across trials.

### Coding and analysis

We recorded participants' behavior and interactions with three video cameras (Panasonic, NV-GS150). The main target event was tool transfer between paired participants. We categorized tool transfer events into three types: “tolerated-theft transfer”, “upon-request transfer”, and “voluntary transfer”. In tolerated-theft transfer, a tool was taken away by the recipient from the giver's hand or mouth, although the giver did not show any facilitation; that is, the giver did not displace the tool toward the recipient. In upon-request transfer, the giver transferred a tool to the recipient upon the recipient's request, for example, poking an arm through the hole between the two booth, vocalizing (including whimpering and screaming), clapping hands, and/or beating the panel between the two booths. In voluntary transfer, the giver actively transferred a tool to the recipient without the recipient's explicit request. When discussing the occurrence of request within pairs across trials, we considered a single data point per trial, i.e. whether or not any request behavior had been recorded during the trial. When a participant turned his or her face toward the partner whilst he or she was attempting to obtain the juice reward without the use of a tool, we counted this behavior as “observation”.

For statistical analysis of the data, we used nonparametric tests which are insensitive to normal distribution and/or data independence. In order to account for the fact that some subjects were used in different pairs, we used random permutation tests, and calculated *p*-value based on 10,000 permutations. We treated separately data from the same participants with different partners. Each adult female in non-kin pairs was paired with two different partners; for example, Pendesa was paired with Puchi and also with Mari. We counted Pendesa's data as two samples. Therefore, the sample size of individuals in non-kin adult pairs was six, and the total sample size was 12.

## Results

### Experiment 1

Tool transfer was observed frequently in the mismatched condition, but seldom in the matched condition. Tool transfer was recorded on average 59.0% (SD = 35.5) of the trials in the mismatched condition, while on average 0.3% (SD = 0.1) of the trials in the matched condition. There was a significant difference between the two conditions (Wilcoxon singed rank test: *N* = 12, *Z* = −2.95, *p* = 0.003). In the matched condition, tool transfer was observed only once from Pal (offspring) to Pan (mother).

**Figure 2:**
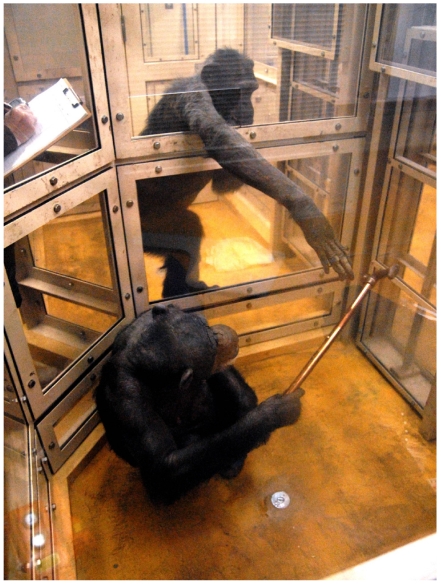
Tool transfer upon recipient's request. A chimpanzee (Mari) in the near-side booth picks up a stick and hands it over to her partner (Pendesa) in the far-side booth who requested the tool by poking her arm through the hole between the booths.

**Figure 3:**
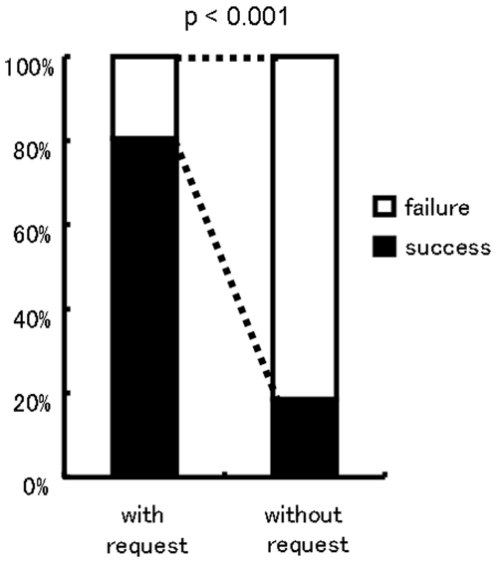
The percentage of recipients' success in receiving a tool from a partner. “with request” is cases in trials in which the recipients exhibited request, and “without request” is cases in trials in which the recipients failed to exhibit request. The data from all the participants were pooled.

Tool transfer occurred mostly following a recipient's request (Figure 2; also see Movie S1 for supporting video). In the mismatched condition, a total of 170 tool transfer events were observed. Upon-request transfer accounted for 74.7% of the events, while voluntary transfer 14.7% and tolerated-theft transfer 10.6% (Table 1). Pooled analysis showed that recipients succeeded in obtaining a tool from their partner in 81.8% (144/176) of total trials in which the recipients demonstrated requesting behavior, while they succeeded in only 23.2% (26/112) of total trials in which they did not exhibit request (Figure 3). There was a significant difference between the two ratios (Chi-square test: *χ* ^2^ (1) = 97.2, *p*<0.001).

**Table 1:** The number of trials during which tool transfer occurred in experiment 1 (24 trials per subjects).

	Ai-Ay		Ch-Cl		Pn-Pl		Pe-Pu		Pe-Ma		Pu-Ma	
	M	O	M	O	M	O	D	S	D	S	D	S
tool transfer	24	21	19	19	19	20	3	0	6	20	3	16
tolerated theft	0	1	2	2	4	5	0	0	0	2	0	2
upon request	23	13	17	14	14	14	0	0	0	18	0	14
voluntary	1	7	0	3	1	1	3	0	6	0	3	0

The first line represents the pairs (Ai: Ai, Ay: Ayumu, Ch: Chloe, Cl: Cleo, Pn: Pan, Pl: Pal, Pe: Pendesa, Pu: Puchi, Ma: Mari). The second line represents the relationship between the pairs (M: mother, O: offspring, D: dominant, S: subordinate). The third line represents the overall number of trials in which a participant transferred a tool to the partner. The remaining three lines below represent the number of each type of tool transfer observed.

Observation of partner's struggle to obtain the juice without the use of a tool (only observed in the stick tool situation) did not elicit voluntary tool transfer. Stick possessors observed the partner reach out for a juice container without a stick in 47 trials, and of these 47 trails, voluntary transfer occurred 7 (14.9%) times; in other words, at a similar rate as that recorded overall (14.7%) (Chi-square test: *χ* ^2^ (1) = 0.001, *p* = 0.97). Since no participant tried to obtain the juice without a tool in the straw-use situation, we could not conduct this analysis for straw transfer.

There were some differences between mother-offspring pairs and non-kin adult female pairs. Tool-transfer events occurred significantly more frequently in the mother-offspring pairs than in the non-kin pairs. Participants gave a tool to a partner on average 84.7% (SD = 8.2) of trials during the mismatched condition in the mother-offspring pairs, while 33.3% (SD = 33.6) in the non-kin pairs (random permutation test: *N*_mother-offspring_ = *N*_non-kin_ = 6, *T* = 51.4, *p* = 0.011). Request was also observed significantly more frequently in the mother-offspring pairs than in the non-kin pairs. Participants exhibited request on average 88.9% (SD = 12.3) of trials during the mismatched condition in the mother-offspring pairs, while 37.5% (SD = 42.7) in the non-kin pairs (random permutation test: *N*_mother-offspring_ = *N*_non-kin_ = 6, *T* = 51.4, *p* = 0.034).

Although social relationship and affiliation may have some influence on performance as suggested in previous studies [34]–[35], partner's request seems nevertheless to be a determining factor. In the mother-offspring pairs, both tool transfer and request were observed bilaterally. The mothers gave a tool to their offspring on average 86.1% (SD = 12.0) of trials during the mismatched condition, and the offspring to their mother on average 83.3% (SD = ±4.2). There was no significant difference in tool transfer frequency between mothers and offspring (Chi-square test for the pooled data from the three mother-offspring pairs: *χ* ^2^ (1) = 0.21, *p* = 0.64). The mother exhibited request toward their offspring on average 84.7% (SD = 15.8) of trials, and the offspring toward their mother on average 93.1% (SD = 8.7). In addition, there was no significant difference between mothers and offspring in their request frequency (Chi-square test for the pooled data from the three mother-infant pairs: *χ* ^2^ (1) = 2.5, *p* = 0.11). In contrast, in the non-kin pairs, tool transfer occurred predominantly from the subordinate individual to the dominant individual and request was mostly observed in dominant individuals. Subordinates transferred a tool to their dominant partner on average 50.0% (SD = 44.1) of trials during the mismatched condition, whereas the dominants transferred a tool to their subordinate partner on average only 16.7% (SD = 7.2) of trials. The frequency of tool transfer was significantly greatly from subordinate to dominant than the reverse (Chi-square test for the pooled data from the three non-kin pairs: *χ* ^2^ (1) = 18.0, *p*<0.001). The dominants exhibited request toward the subordinate partner on average 63.9% (SD = 48.1) of trials, whereas the subordinates toward the dominant partner on average 11.1% (SD = 12.7). Dominant individuals requested significantly more frequently than did subordinates (Chi-square test for the pooled data from the three non-kin pairs: *χ* ^2^ (1) = 42.8, *p*<0.001).

### Experiment 2

Table 2 shows the number of trials in which a tool was transferred to the partner during the course of the 24 trials conducted in experiment 2. Overall, tool transfer was observed on average 90.3% (SD = 12.3) of the trials. The percentage of tool transfer was the same whether from mother to offspring (average = 90.3%, SD = 9.6) or from offspring to mother (average = 90.3%, SD = 16.8) (Chi-square test for the pooled data from the three mother-offspring pairs: *χ* ^2^ (1) = 0.0, *p* = 1.0) There was no significant difference in the tool transfer rate in the individuals of the mother-offspring pairs between experiment 1 and 2 (Wilcoxon signed rank test: *N* = 6, *Z* = −0.94, *p* = 0.35). In order to investigate whether tool transfer was initially maintained due to the immediate reciprocal context previously encountered in experiment 1, we compared the first 12 trials with the last set of 12 trials we conducted in experiment 2. There was no significant difference in the tool transfer rate between the first 12 trials and the second 12 trials (Wilcoxon signed rank test: *N* = 6, *Z* = −1.13, *p* = 0.26). The latency before receiving a tool was also not significantly different between the two sets of trials (we counted 300 sec for the trials in which a tool was not transferred; Wilcoxon signed rank test: *N* = 6, *Z* = −0.10, *p* = 0.92).

**Table 2:** The number of trials in which tool transfer was recorded in experiment 2 (24 trials).

	Ai-Ay		Ch-Cl		Pn-Pl	
	M	O	M	O	M	O
tool transfer	24	21	19	19	19	20
tolerated theft	0	1	2	2	4	5
upon request	23	13	17	14	14	14
voluntary	1	7	0	3	1	1

See Table 1 legend for explanation of the table. The difference between experiment 1 and 2 is that the role of donor and recipient was fixed in experiment 2 for 24 successive trials, approximately for one week, during which time participants in the donor's role could not receive short-term reciprocation from their partner.

In experiment 2, as well as in experiment 1, tool transfer occurred mostly following a recipient's request. A total of 130 tool-transfer events were observed. Upon-request transfer accounted for 75.4% of the events, while voluntary transfer 7.7% and tolerated-theft transfer 16.9%. We compared the ratios of the types of tool transfer in the mother-offspring pairs between experiment 1 and 2, and found no significant difference (Chi-square test: *χ* ^2^ (2) = 2.8, *p* = 0.25).

## Discussion

The present study demonstrates that chimpanzees can instrumentally help a conspecific partner by transferring a tool which the partner needs to solve a task. The chimpanzees in experiment 1 demonstrated tool transfer in the mismatched condition but seldom in the matched condition where tool transfer was unnecessary for obtaining the juice reward. The participants thus transferred a tool to their partner when the partner needed the tool but rarely in other contexts such as play. This helping behavior was observed not only in mother-offspring pairs but also in non-related adult pairs. The present study adds to the experimental evidence of chimpanzees' altruism toward conspecifics, reported so far in only one experimental setup (experiment 3 of Warneken et al.'s study [14]).

The tool transfer experimental paradigm used in this study might have favored the expression of altruism in the chimpanzee subjects, since transfer involved objects rather than foods (although the tool was then subsequently used to obtain a food reward) and the set-up permitted the expression of request behavior(s) between partners. The chimpanzee mothers and offspring in the present study gave a tool to a partner 84.7% of the trials in experiment 1 and 90.3% in experiment 2. In contrast, in a previous study [25], which experimentally examined food sharing from the same mothers to offspring (when the offspring were less than 2 years old), food was transferred only 32.0% of the trials. This comparison supports the idea that altruism in chimpanzees can be seen in situations not involving food [14]–[15], [19], and that the experimental paradigm in the present study using tools is more appropriate in eliciting prosocial tendency in chimpanzees.

The point of our study was to investigate proximate factors for chimpanzees' targeted helping rather than ultimate factors such as inclusive fitness. The results from the present study emphasize the importance of request as a communicative interaction between chimpanzees. In most cases, request was demonstrated in the form of poking an arm through the hole, which was obviously directed at a potential helper. The other forms of request such as vocalizing, clapping hands, and beating the panel, were often performed in combination with arm poking and/or placing a hand at the entrance of the hole and/or looking at a potential helper, which additionally indicated that the request was directed at the conspecific partner. Request increased recipients' success in receiving a tool, and participants seldom transferred a tool voluntarily to their partner in the absence of request behavior. Not only between a chimpanzee and a human [7]–[8], but also between chimpanzees themselves, request therefore appears to act as an important communicative behavior prompting targeted helping.

These observations support the idea that chimpanzees more readily perform recipient-initiated altruism than voluntary altruism [19]. In the previously mentioned experimental study investigating food sharing from mother to infant [25], voluntary (mother-initiated) food sharing was actually absent. Considering that chimpanzees' food sharing in the wild also arises predominantly following recipient's initiative [12], [36], recipient-initiated altruism seems to characterize at least food or object sharing events in chimpanzees, and is not therefore restricted to the experimental paradigm we employed in the present study.

Request rather than short-term reciprocity seems to be a more important mechanism in explaining chimpanzees' targeted helping behavior. Tool transfer continued predominantly following a recipient's request not only in experiment 1 but also in experiment 2 in which the participants could not expect any short-term reciprocation from their partner. In the latter experiment, the tool transfer rate did not decrease across trials, although the participants assigned as givers did not receive any reciprocation from their partner continuously for 24 trials for approximately one week. Although we could not rule out the role of long-term reciprocation, chimpanzees' targeted helping behavior was not in this case maintained by short-term reciprocation. Alternated bi-directional request behavior might act as a mechanism enabling the establishment and maintenance of reciprocity between partners (e.g. individual A responds to individual B's request, and thereafter individual B responds to individual A's request). Previous studies which reported reciprocity in chimpanzees [37]–[39] and other non-human primates [40]–[43] have unfortunately not fully paid attention to recipients' behavior nor communicative interaction. A simple reciprocal experimental paradigm which prevented direct physical interaction between participants failed to elicit chimpanzees' other-regarding preferences, reciprocal cooperation [22] and spontaneous barter [44]. Chimpanzees are therefore more likely to help others upon recipient's request than on a voluntary basis or based on short-term reciprocity.

The remaining question is whether or not high frequency of voluntary altruism is unique to humans. The fact that some new world monkeys (common marmoset [7], capuchin monkey [8]–[9], cotton-top tamarin [43], [but see also 45]) have demonstrated unsolicited prosociality suggests that voluntary altruism evolved in phylogenetically diverse taxa. As for now, there seems to be no consensus on what is the decisive factor in explaining species differences. Cooperative breeding, for example, was considered as one of the candidates [7]; however, capuchins, non-cooperative breeder, showed unsolicited prosociality [8]–[9], and cotton-top tamarins, cooperative breeders, did not in a similar experimental context [45]. Social relationship such as tolerance [34]–[35] and/or dominance relationship as suggested in the present study might be a more plausible influential factor. Further detailed studies investigating the social and/or ecological selective pressures favoring or hampering prosocial propensity in animals should lead us to a better understanding of the evolution of altruism.

In conclusion, chimpanzees as well as humans can help others without pursuing personal benefits. However in contrast to humans, chimpanzees help each other upon request but seldom voluntarily. In the present study, even when the chimpanzees observed their conspecific partner unsuccessfully struggle to reach the juice container without a stick tool, the tool possessor often failed to offer the tool voluntarily unless explicitly requested. The rarity of voluntary targeted helping behavior in chimpanzees is worth further attention and investigation. The fatal obstacle might be chimpanzees' imperfect understanding of others' desires, which is possibly derived from a lack of understanding of other's beliefs [46], understanding of triadic relationships [47], and/or of active teaching [48]. Although chimpanzees appear to share some important aspects of altruism with humans, it is also possible that the evolution of altruism followed different paths and experienced different selective pressures after the split between *Homo* and *Pan*.

## Supporting Information

Movie S1 Tool transfer upon recipient's request. In this scene in experiment 1, Mari (right booth) was in the straw-use situation and was supplied with a stick. Puchi (left booth) was in the stick-use situation and was supplied with a straw. After a human experimenter supplied a stick and a straw from the ceiling, Puchi poked her arm through the hole, and Mari responded to Puchi's request and gave a stick to her. And then Puchi got juice. Mari observed Puchi to drink juice, but did not demonstrate request. No transfer from Puchi to Mari occurred in this session.
(5.99 MB MOV)
