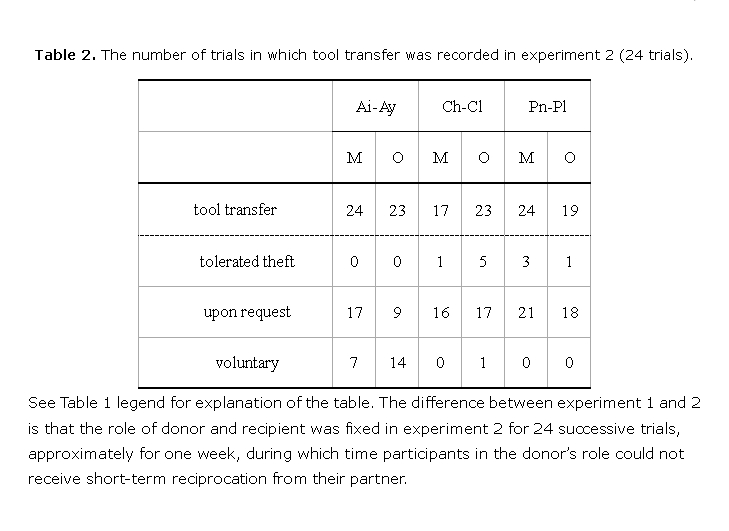# Correction: Chimpanzees Help Each Other upon Request

**DOI:** 10.1371/annotation/80db4649-46c1-40af-851b-f01968eec5d7

**Published:** 2009-10-27

**Authors:** Shinya Yamamoto, Tatyana Humle, Masayuki Tanaka

The data in Table 2 are duplicated from Table 1. Please view the corrected Table 2 here: 

**Figure:**